# Applicability of the assessment of chronic illness care (ACIC) instrument in Germany resulting in a new questionnaire: questionnaire of chronic illness care in primary care

**DOI:** 10.1186/1472-6963-11-164

**Published:** 2011-07-07

**Authors:** Jost Steinhaeuser, Katja Goetz, Dominik Ose, Katharina Glassen, Iris Natanzon, Stephen Campbell, Joachim Szecsenyi, Antje Miksch

**Affiliations:** 1 Department of General Practice and Health Services Research, University of Heidelberg, Voßstraße 2, Heidelberg 69115, Germany; 2 Health Sciences - Primary Care Research Group, University of Manchester, Williamson Building Oxford Road, Manchester M13 9PL, UK

## Abstract

**Background:**

The Chronic Care Model (CCM) is an evidence based, population based approach to improve care for people with chronic conditions. The Assessment of Chronic Illness Care (ACIC) instrument is widely used to measure to what extent within a healthcare system the CCM is implemented. The aim of this study was to translate and culturally adapt the ACIC Instrument for the German healthcare system.

**Methods:**

For translating the ACIC instrument, principles of Good Practice for the Translation and Cultural Adaptation Process by the ISPOR Task Force were followed. Focus groups were additionally conducted with general practitioners to adapt the items culturally.

**Results:**

The ACIC instrument can not be used in the German healthcare system easily due to a multifaceted understanding of words, different levels of knowledge of the CCM and fundamental differences between health systems.

**Conclusions:**

As following the CCM leads to benefits for patients with chronic illnesses, measuring to which extent it is implemented is of major interest. A new questionnaire using the CCM as its theoretical basis, sensitive to the healthcare systems of the host country has to be created. Knowledge transfer between countries by using an instrument from a different healthcare system can lead to a completely new questionnaire.

## Background

Worldwide, the number of patients with chronic diseases and multiple conditions is growing [1]. Multimorbidity refers to the co-occurrence of two or more chronic conditions [2] and affects people's quality of life, utilization of healthcare systems and mortality [3,4]. There is quite little evidence about how to treat multimorbid patients [5]. The Chronic Care Model (CCM) is an evidence based approach for improving care for patients with chronic conditions [6]. It was developed in the United States of America (USA) during the 1990s and was designed to improve patient health outcomes by making changes in the way care is provided [7]. The CCM has proven benefits for chronically ill patients [6]. Therefore, its implementation in primary care seems to be promising according to the German Advisory Council on the Assessment of Developments in the Healthcare System [8].

The CCM consists of six interrelated components: organization of healthcare delivery system and community linkages at the system level, self management support, delivery system, decision support and clinical information systems at the practice level. The Assessment of Chronic Illness Care (ACIC) was developed to measure the degree to which a healthcare system adheres to elements of the CCM [9]. However none of these studies were performed in Germany. The elements of the CCM as well as the integration effect that occurs when all model elements are engaged can be measured using ACIC. Although the ACIC instrument was not formally validated, it has been used extensively in quality improvement programs where it displayed face validity for the CCM and has been shown to be sensitive to change.

The US and German healthcare systems differ in several aspects. While Germany has a social insurance system for about 90% of all patients, the USA has a market economy system for about 72% of all patients. Different from many other countries the majority of practices in Germany are small and single handed. The CCM was first introduced in Germany in 2006 [10-12]. The only assessments of its implementation are from the patient's point of view, as determined using the Patient Assessment of Chronic Illness Care (PACIC), a patient-centered outcome instrument based on the CCM [13-15]. As this instrument focuses on the patient's point of view of care and does not have items concerning the healthcare delivery system, it was feasible to use it in former research projects outside of the USA [16].

A group of researchers at the Department of General Practice and Health Services Research at the University Hospital Heidelberg (Germany) aimed to translate and culturally adapt the ACIC instrument.

## Methods

### Participants

To recruit GPs for focus groups we invited by fax 250 teaching practices of the University Hospital Heidelberg (Germany) and in addition 80 GPs meeting in two different quality circles [17] from the area of Stuttgart.

### Procedures

The ACIC instrument measures changes in the following domains: organization of healthcare delivery system, community linkage, self management support, decision support, delivery system, clinical information system and integration of CCM. In these domains participants can rate 0 (D level: worst) up to 11 (A level: best) for each item.

To adapt the ACIC instrument for German healthcare settings we followed the Principles of Good Practice for the Translation and Cultural Adaptation Process by the ISPOR task force [18] as follows:

1. We received permission from the authors of the ACIC Instrument at the MacColl Institute (USA), to develop a German version of the instrument.

2. In Spring 2008 we simultaneously conducted two different forward translations using two different teams.

3. After that a consensus forward translation was discussed by all health services researchers of our department involved in the process of translation.

Queries about the meaning of specific items in the questionnaire were clarified by a member of the MacColl Institute. The product of the consensus process, the consensus version, is available in German in the Additional file 1. Furthermore, items should be culturally adapted through focus groups (FG) with general practitioners (GP). The two FG sessions lasted between 120-150 minutes. FGs were conducted by two researchers. The structured guideline for the FG was to answer questions concerning:

a) whether an item was understandable,

b) whether the content of an item was important concerning care of chronically ill patients and

c) whether there were any important aspects missing regarding care of the chronically ill in Germany.

In order to ensure comparability between groups, we asked the same questions of both.

The FG were recorded verbatim by audio and video, fully transcribed and analyzed separately by two researchers. A categorizing system was developed based on the structured guideline for the FG [19,20].

## Results

### Characteristics of the study sample

For the composition of the translation/consensus version teams, please see table 1.

**Table 1:** Characteristic of translation/consensus version teams (n = 6)

Characteristics	
sex female/male	4/2
age; mean (SD); range	33.43 (4.04); 28-41
profession	
physicians	3
health professional	1
sociologists	2

SD standard deviation

From the 330 invited GPs, 25 GPs expressed an interest in participating, though only nine actually did so. The reasons given by the 16 who did not show up were: "lack of time" and "distance of travel to the FG". The other 305 did not reply. Sociodemographic characteristics of participating GPs are shown in table 2.

**Table 2:** Characteristics of GP focus groups (n = 9)

Characteristics	
sex female/male	3/6
age; mean (SD); range	53.88 (6.05); 47-67
office based since; mean (SD); range	20.55 (6.54); 16-36
single handed practice	5
group practice	4
located: city	2
located: outskirts	5
located: countryside	2

SD standard deviation

#### Translation

The process of translation and consensus resulted in an accepted version of the translated ACIC. There were difficulties in understanding the exact meaning of some items of the translated ACIC. This was, apart from the differences in both healthcare systems, due to a lack of understanding of the CCM and an ambiguous phrasing of the items which were not precise enough in German. Furthermore, some of the concepts of the ACIC seemed not as relevant to German healthcare settings.

From the six elements of the CCM, items from "clinical information system", "community linkage" and especially "self management support", were less difficult to understand by the translation team. Whereas items relating to "organization of healthcare delivery system", "decision support" and "delivery system design" were more difficult to understand and to apply to German healthcare settings.

One example of a multifaceted understanding of words was: "Community Linkages". The word "community" is very imprecise as in German it can mean municipality, association or alliance. Therefore this entire part of the ACIC can be understood in many different ways.

A further example is in "Organization of the Healthcare Delivery System": The first ACIC item concerning the overall Organizational Leadership in Chronic Illness Care says at "C level" "*(...) is reflected in vision statements and business plans, but no resources are specifically earmarked to execute the work."*

Our translation group did not understand how "business plans" fits in this context.

What we learned after asking the authors at the MacColl Institute was that healthcare organizations in the USA must support the changes financially at the planning level.

The conclusion of this procedure was that a translated ACIC can not easily be completed by respondents. As such, we had to revise our strategy for its cultural adaptation and the back translation was not performed. We decided to build a completely new questionnaire. The main domains of the consensus version of the ACIC instrument formed one basis of a new questionnaire entitled "Questionnaire of Chronic Illness Care in Primary Care" (QCPC). This entirely new questionnaire consists of relevant aspects of the CCM: Delivery system (12 questions), community linkage (5 questions), self management support (6 questions), decision support (7 questions) and clinical information system (5 questions). Furthermore the QCPC includes socio demographic questions and aspects of chronic illness care relevant to Germany such as structure of practices (23 questions), quality management (3 questions) and disease management programs (2 questions). From our experiences with difficulties in using the ACIC instrument in a different healthcare system, we did not include questions regarding Organisation of healthcare delivery system in the QCPC. For further details regarding differences with the ACIC please see table 3.

**Table 3:** Differences ACIC and QCPC

CCM component	Number of ACIC items	Number of QCPC items
Organization of healthcare delivery system	6	0
Community linkages	3	5
Self management support	4	6
Delivery system	6	12
Decision support	4	7
Clinical information system	5	5

**Categories of Answer**	ACIC	QCPC

	Scores: D Level (0-1) up to A Level (9-11) resulting in a sum score as well as score for the single CCM component used for quality improvement programs.	Five-point-Likert scale e.g. using "always" to "never" or percentage as well as yes/no answers resulting in a tailored feedback of the single practice used for improvement of chronic illness care.

**Directions**	ACIC	QCPC

	Can be filled out by anyone of one physical site (e.g., a practice, clinic, hospital, health plan) that supports care for chronic illness (condition has to be specified).	Should only be filled out by a primary care physician who supports care for patients with chronic illnesses (condition has not to be specified).

As this new questionnaire had to be built, the types of answers to ACIC are different, too. New items were developed according to the rules of questionnaire design by Porst [21].

Focus groups (FG) were now asked to comment on items of QCPC instead of ACIC. Items concerning the CCM were integrated as close to its original version as possible. Categories of answers were changed into yes/no or Likert scales.

#### Focus groups

In the FG some items with a CCM background, were not considered to be important. Example referring to "Delivery System Design"

Physicians saw no need for a system to track patients if they went to an appointment by a specialist or not, or if they got a report or not.

*"(...) you would need to know, if your chronically ill patient is already discharged, or still in hospital. (...) to follow up results I would need to know if my patient is at hospital at all." *(P3)

Example referring to "Decision Support"

Using guidelines was considered to be more like an "add on", something physicians have in mind when treating patients, rather than following guidelines strictly.

"*I notice every guideline in an informative way, I save it in my head, but I will not actually go get the guideline and look something up. (...) I only need it sometimes as an excuse if something happens, meaning recourse, medical recourse or something*." (P5)

### Important gaps

The most important gaps from the point of view of the GPs in the QCPC version were: questions concerning where care is provided, referring especially to home visits. Other questions which should be included would concern how time-consuming different elements of care are and what equipment every practice has to provide (ultrasound, proctoscope, etc.).

*"(...) many patients that have been chronically ill for years have been treated at home as they are immobile; this is a totally different clientele but chronically ill diabetic (...)." *(P2)

*"Almost every practice has an ultrasound machine, aeroplethysmograph, of course ECG - that is not the standard in foreign countries. (...) and the question do you do home visits?" *(P3)

## Discussion

In several cases with a multifaceted understanding of words, the ACIC instrument was not translatable in a way that made items precise enough. Referring to the first of the "ten rules for building a questionnaire" by Porst an item has to be unambiguous and easy to understand [20]. Knowledge transfer between countries by using an instrument from a different health system can not be done without adaptation to the cultural specifics of the country where it should be used. As the organization of the two healthcare systems is different, some items can not be understood the same way, as they pertain more to the USA system.

The ACIC instrument was originally a product from quality improvement collaboratives focusing on care for the chronically ill in the USA [9,22]. The whole process of improving care for chronically ill patients within ACIC is therefore strongly influenced by the needs of USA collaboratives. An instrument resulting out of this process does not necessarily fit into another health system as needs might be different. The ACIC is more a process stimulating tool but a questionnaire and therefore rather educational.

As shown above, "tracking patients" and "guidelines" do not seem to have the same importance in the different health systems compared in this paper. This might be due to the fact that there are far fewer incentives in Germany for using guidelines. Although primary care based Disease Management Programs for chronic conditions are widely implemented in Germany [13], studies performed to examine the preparation of implementation of guidelines in Germany, suffer from low response rates [23].

Differences in understanding the items from the ACIC instrument might to some degree be due to differences in providing care in the two different countries. Continuity of care differs in both countries as patients will not change "their" GP as often in Germany as USA patients do. As many practices in Germany are single handed, physicians might know "their" multimorbid patients for years and not feel to be in need of a register. Cost related access problems are about half as high as in the USA so German physicians might see patients in earlier stages of a disease. Difficulties in getting care after hours without going to the emergency room occur about twice as often in the USA. Moreover this might have consequences on continuity of care. Problems in having former results available at the time of the next appointment happen twice as often in the USA, as in Germany. This might explain why GPs in the FG saw no further need for tracking patients. Consistent with the statements of our FG, discharge gaps occur less in the USA [24].

In literature about transferring quality indicators from van der Ploeg et al. there is another good example of limitation by a knowledge transfer process from one country to another. One third of these quality indicators could not be transferred from the USA to a European country [25]. As there is good evidence for implementing elements of the CCM to improve care for the chronically ill [26], a questionnaire measuring baseline and changes in such care as the ACIC instrument does, has to be created in the country where it is to be used. Limitations of poor international comparability should be considered less important than starting from the wrong baseline. There are two alternatives seen in literature; the first is to use a scoring team familiar with the ACIC instrument [27] and the second is to try to capture the original idea of an item [28]. While the first can not be used in a high number of practices and has a risk for potential bias, the second could be used by a managed care organization. However managed care organizations are at the planning level yet in Germany.

Compared to the small number of GPs who could be recruited for this study, the fraction of GPs working in a group practice is overrepresented as in Germany single handed practices are still most common. Furthermore selection bias is an important limitation of our study as only highly motivated GPs did participate in the FG [29].

## Conclusions

In our experience a cultural adaptation of the ACIC instrument close to the original version is impossible. A questionnaire measuring baseline and changes in such care as the ACIC instrument does, has to be created in the country where it is to be used. Different from the ACIC instrument, the QCPC can be used by primary care physicians without being teached in the core elements of the CCM before. The next step for the QCPC questionnaire will be the validation study. In the future, the QCPC will be used within the ESTHER cohort, including about 600 GPs and 10,000 patients, to see whether structured care according to the CCM leads to better care of multimorbid (and frail) patients [30,31]. Each participating GP will get an individualised feedback of the QCPC results, underlining fields of improvement for reasons of benchmarking.

## Competing interests

The authors declare that they have no competing interests.

## Authors' contributions

All authors made substantive contributions to the study. JS, KG, DO, KGl, IN, JSz, and AM contributed in producing a consensus version of the ACIC instrument and in further conception of the QCPC. SC has been involved in critical revising the manuscript.

All authors read and approved the final manuscript.

## Pre-publication history

The pre-publication history for this paper can be accessed here:

http://www.biomedcentral.com/1472-6963/11/164/prepub

## Supplementary Material

Additional file 1 **The result of the consensus version of the translated *Assessment of Chronic Illness Care *instrument**. Tables of the consensus version of the translated *Assessment of Chronic Illness Care *instrument in German language. Click here for file
