# Supplementary material for: Applicability of the assessment of chronic illness care (ACIC) instrument in Germany resulting in a new questionnaire: questionnaire of chronic illness care in primary care
Source: BMC Health Serv Res. 2011 Jul 7;11:164. doi: 10.1186/1472-6963-11-164 (PMC3145559; doi:10.1186/1472-6963-11-164)
Supplement: Additional file 1 — The result of the consensus version of the translated Assessment of Chronic Illness Care instrument. Tables of the consensus version of the translated Assessment of Chronic Illness Care instrument in German language. [file 1472-6963-11-164-S1.DOC]

**Result of the consensus version of the translated Assessment of Chronic Illness Care (ACIC) instrument**

| **Teil 1: Die Organisation des Gesundheitssystems.** Die strukturierte Versorgung chronisch Kranker kann  effektiver werden, wenn im gesamten Gesundheitssystem der Schwerpunkt darauf gelegt wird. |
| --- |

| **Übergeordnete Zuständigkeiten zur Versorgung chronisch Kranker** | … sind nicht vorhanden oder haben wenig Bedeutung | … spiegeln sich in Zielstellungen und Vorgaben wieder, aber es sind keine spezifischen Ressourcen für die Umsetzung vorgesehen | … spiegeln sich in der oberen Führungsebene und speziell zugeordneten finanziellen und personellen Mitteln wieder | …sind Teil der langfristigen Planungsstrategie der Organisation. Finanzielle Mittel stehen zur Verfügung und verantwortliche Personen sind benannt. |
| --- | --- | --- | --- | --- |

| **Übergeordnete Ziele in der Versorgung chronisch Kranker** | … gibt es nicht oder sind nur auf einen Aspekt begrenzt. | … gibt es, werden aber nicht überprüft. | … sind messbar und werden überprüft. | … sind messbar, werden routinemäßig überprüft und werden in Maßnahmen zur Verbesserung eingebunden |
| --- | --- | --- | --- | --- |

| **Gesamtkonzept zur Verbesserung der Versorgung chronisch Kranker** | … ist weder vorausschauend noch kontinuierlich organisiert. | … entspricht spontan entwickelten Lösungsansätzen. | … verwendet bewährte Lösungsstrategien, wenn Probleme auftreten. | … beinhaltet eine bewährte Lösungsstrategie und verwendet sie vorausschauend um die übergeordnete Ziele zu erreichen. |
| --- | --- | --- | --- | --- |

| **Anreize und Vorgaben für die Versorgung chronisch Kranker** | … werden nicht verwendet um Versorgungsziele zu beeinflussen. | … werden genutzt um die Inanspruchnahme und Kosten der Versorgung chronisch Kranker zu beeinflussen. | … werden genutzt um die Ziele der Versorgung der Patienten zu unterstützen. | … werden genutzt um Leistungserbringer zu motivieren und zu qualifizieren, um die Ziele der Versorgung der Patienten zu unterstützen |
| --- | --- | --- | --- | --- |

| **Führungsebene** | … lehnt die Aufnahme von chronisch Kranken ab. | … räumt der Verbesserung der Versorgung chronisch Kranker keine Priorität ein. | … ermutigt zur Verbesserung der Versorgung chronisch Kranker. | … unterstützt sichtbar die Verbesserung der Versorgung chronisch Kranker. |
| --- | --- | --- | --- | --- |

| **Anreize für Patienten** | … verhindern eine Veränderung des Selbstmanagements oder des Versorgungssystems. | … führen weder zu einer Förderung noch Verhinderung von Veränderungen des Selbstmanagements oder des Versorgungssystems. | … fördern Veränderungen des Selbstmanagements oder des Versorgungssystems. | … werden speziell danach ausgerichtet, um die Versorgung chronisch Kranker zu verbessern. |
| --- | --- | --- | --- | --- |

| **Teil 2*:* Kommunale Vernetzungen.** Die Vernetzung zwischen dem Gesundheitssystem und den kommunalen Strukturen spielt eine wichtige Rolle bei der Versorgung chronisch Kranker. |
| --- |

| **Vernetzung der Patientenversorgung mit externen Einrichtungen** | …ist nicht vorhanden | …beschränkt sich auf eine Auswahl kommunaler Angebote | …wird durch eine ausgewiesene Person durchgeführt. Diese ist verantwortlich, den maximalen Nutzen aus den vorhandenen Ressourcen für Leistungserbringer und Patient zu erreichen. | …zeichnet sich durch eine aktive Koordination zwischen dem Gesundheitssystem, den kommunalen Strukturen und den Patienten aus. |
| --- | --- | --- | --- | --- |

| **Zusammenarbeit mit kommunalen Organisationen** | …ist nicht vorhanden | …wurde erwägt jedoch noch nicht umgesetzt. | …wurde begonnen, um unterstützende Programme und Konzepte zu entwickeln | ..wird aktiv dazu genutzt, um unterstützende Programme und Konzepte für das gesamte System weiterzuentwickeln |
| --- | --- | --- | --- | --- |

| **Regionale Gesundheitskonzepte** | …koordinieren nicht bestehende Leitlinien, Maßnahmen oder Ressourcen für die Versorgung chronischer Erkrankungen auf Ebene der Leistungserbringer | …beinhaltet ein gewisses Maß an Koordination von Leitlinien, Maßnahmen oder Ressourcen zur Versorgung chronischer Erkrankungen. Diese hat auf der Ebene der Leistungserbringer noch zu keiner Veränderung geführt. | .. beinhaltet die Koordination von Leitlinien, Maßnahmen oder Ressourcen zur Versorgung von ein oder zwei chronischen Erkrankungen. | …beinhaltet die Koordination von Leitlinien, Maßnahmen oder Ressourcen zur Versorgung der meisten chronischen Erkrankungen. |
| --- | --- | --- | --- | --- |

| **Teil 3: Ebene der Leistungserbringer.** Die Einführung einzelner Elemente auf der Ebene der Leistungserbringer (z.B. individuelle Praxen) hat zur Verbesserung der Versorgung chronisch Kranker geführt. Diese sind: Unterstützung des Selbstmanagements, Verbesserung der Praxisstruktur, Entscheidungshilfen und klinische Informationssysteme. |
| --- |

**Teil 3a: Unterstützung des Selbstmanagements.** Eine effektive Unterstützung des Selbstmanagements kann Patienten und deren Familien helfen, mit den Herausforderungen eines Lebens mit chronischen Erkrankungen und deren Behandlung zurechtzukommen. Zudem können Komplikationen und Symptome reduziert werden.

| **Dokumentation und Beurteilung von Bedürfnissen und Aktivitäten des Selbstmanagement** | … wird nicht gemacht | … ist geplant | … existiert in standardisierter Form | … wird regelmäßig durchgeführt und fließt in standardisierter Form in einen Behandlungsplan ein, der für Praxen und Patienten verfügbar ist. |
| --- | --- | --- | --- | --- |

| **Unterstützung des Selbstmanagements** | … ist beschränkt auf die Verbreitung von Informationsmaterial (Broschüren, Hefte) | … findet durch die Empfehlung/ Überweisung an eine Gruppenschulung oder einen Berater statt. | … wird durch eine eigens ernannte, entsprechend ausgebildete Person durchgeführt. Diese kann ein Mitarbeiter der Praxis oder an die Praxis angegliedert sein und sieht Patienten durch Empfehlung/ Überweisung. | … wird durch eine Person durchgeführt, die speziell dafür ausgebildet ist, beim Patienten Empowerment/ Selbstfürsorge und Problemlösekompetenz zu stärken. Diese kann ein Mitarbeiter der Praxis oder an die Praxis angegliedert sein und sieht die meisten Patienten mit chronischer Erkrankung. |
| --- | --- | --- | --- | --- |

| **Berücksichtigung der Anliegen von Angehörigen** | … findet nicht immer statt. | … wird nur für spezielle Patienten und ihren Familien durch Überweisung ermöglicht. | … wird gefördert, Selbsthilfegruppen und Beratungsprogramme stehen zur Verfügung. | … ist ein integraler Bestandteil der Behandlung und beinhaltet systematische Beurteilung und routinemäßiges Einbeziehen von Selbsthilfegruppen oder Beratungsprogramme |
| --- | --- | --- | --- | --- |

| **Maßnahmen zur Unterstützung von Verhaltensänderung und Selbsthilfe.** | … finden nicht statt. | … sind begrenzt auf die Verteilung von Broschüren, Heften, oder anderen schriftlichen Informationen. | … sind nur durch die Überweisung an spezielle Zentren mit geschultem Personal zugänglich. | ... sind leicht zugänglich und ein integraler Bestandteil der Routineversorgung. |
| --- | --- | --- | --- | --- |

**Teil 3b: Entscheidungshilfen**. Effektive Programme zur Versorgung chronisch Kranker ermöglichen, dass die Leistungserbringer Zugang zu evidenzbasierten Informationen zur Unterstützung der Entscheidungsfindung und Therapie bekommt. Diese beinhalten evidenzbasierte Praxis-Leitlinien oder Protokolle, Einbezug von Fachärzten, Fortbildungen und Patienteninformationen.

| **Evidenzbasierte Leitlinien** | …sind nicht verfügbar. | …sind verfügbar jedoch nicht in die Versorgung implementiert. | …sind verfügbar. Die Implementierung wird durch Schulung der Leistungserbringer unterstützt. | …sind verfügbar. Die Implementierung wird durch Schulung der Leistungserbringer und den Einsatz von Remindern und anderen Maßnahmen zur Optimierung der Versorgung unterstützt. |
| --- | --- | --- | --- | --- |

| **Einbindung von Experten/ Fachärzten zur Verbesserung der Primärversorgung** | …vor allem durch Überweisung. | … hat das Ziel, den Wirkungsgrad der Leitlinien-Implementierung im gesamten Versorgungssystem zu erhöhen. | …beinhaltet Schulungen für Praxisteams durch Experten/ Fachärzte. | …beinhaltet eine kontinuierliche interdisziplinäre Zusammenarbeit zur Verbesserung der Primärversorgung der Patienten. |
| --- | --- | --- | --- | --- |

| **Fortbildung der Leistungserbringer für die Versorgung chronisch Kranker.** | …wird unregelmäßig angeboten. | …wird regelmäßig durch traditionelle Methoden (z.B. Frontalunterricht) angeboten. | …wird durch die Verwendung optimaler Methoden angeboten. | …beinhaltet eine Fortbildungen des gesamten Praxisteams, wie z.B. der Umgang mit speziellen Patientengruppen oder die Unterstützung des Selbst-Managements. |
| --- | --- | --- | --- | --- |

| **Informieren der Patienten über Leitlinien** | …findet nicht statt. | …findet auf Nachfrage oder durch allgemeine Veröffentlichungen statt. | …erfolgt durch spezifisches Informationsmaterial für jede Leitlinie. | …beinhaltet spezifische Materialien, die für Patienten entwickelt wurden, um ihre Rolle in der Erreichung der Leitlinien-Adherence zu beschreiben. |
| --- | --- | --- | --- | --- |

**Teil 3c:** **Gestaltung der Leistungserbringung.** Es gibt Belege dafür, dass eine erfolgreiche Versorgung chronisch Kranker mehr beinhaltet, als nur zusätzliche Elemente zu dem herkömmlichen, auf die Akutversorgung fokussierten System hinzuzufügen. Vielmehr sind Änderungen in der Praxis-Organisation notwendig.

| **Ausrichtung der Teamstruktur an der Versorgung chronisch Kranker** | …wird nicht berücksichtigt. | …wird durch das Vorhandensein von Personen gewährleistet, die eine angemessene Schulung erhalten haben. | … wird durch regelmäßige Teambesprechungen gewährleistet. Diese beschäftigen sich mit Leitlinien, Zuständigkeiten und Problemen. | …wird durch Teams gewährleistet, die sich regelmäßig treffen und klar definierte Aufgaben haben. Diese beinhalten Patientenschulung in Selbst-Management, proaktive Verlaufskontrolle, Koordination der Hilfsmittel und andere Qualifikationen. |
| --- | --- | --- | --- | --- |

| **Leitung des Teams** | …wird weder intern noch extern wahrgenommen. | …wird von einer übergeordneten Instanz übernommen, um bestimmte Organisationsaufgaben wahrzunehmen | …ist durch die Ernennung eines Teamleiters gewährleistet. *Die Aufgaben im Team sind nicht definiert.* | ..wird durch einen Teamleiter sichergestellt, der Aufgaben und Verantwortlichkeiten festlegt. |
| --- | --- | --- | --- | --- |

| **System zur Terminplanung** | …kann genutzt werden, um Akut-Hausbesuche und Verlaufskontrollen zu planen. | …gewährleistet eine geplante Verlaufskontrolle bei chronisch kranken Patienten. | …ist flexibel und kann individuell angepasst warden. | …beinhaltet eine strukturierte Versorgung, die es dem Patienten ermöglicht, an einem Termin interdisziplinär behandelt zu werden. |
| --- | --- | --- | --- | --- |

| **Verlaufskontrolle** | …wird von Patienten oder Leistungserbringer spontan anberaumt. | …wird durch die Praxis gemäß der Leitlinien geplant. | …wird durch das Praxisteam auf Grundlage der Kontrolle der dokumentierten Patientenkontakte sichergestellt. | …wird individuell an die Bedürfnisse der Patienten angepasst und persönlich, telefonisch oder per E-Mail durchgeführt. Die Vorgaben der Leitlinien werden berücksichtigt. |
| --- | --- | --- | --- | --- |

| **Geplante Konsultationen zur Versorgung chronisch Kranker** | …finden nicht statt. | …finden gelegentlich für Patienten mit schwierigen Verläufen statt. | …werden für interessierte Patienten angeboten. | …findet für alle Patienten statt und beinhalten regelmäßige Untersuchungen und Beurteilungen, Vorsorgemaßnahmen und Unterstützung des Selbst-Managements. |
| --- | --- | --- | --- | --- |

| **Kontinuität der Versorgung** | …ist nicht gewährleistet. | …hängt von der schriftlichen Kommunikation zwischen Haus- und Fachärzten und sonstigen Leistungserbringern ab. | …hat zwischen Haus-, Fachärzten und sonstigen Leistungserbringern einen hohen Stellenwert, ist jedoch nicht systematisch gewährleistet. | …hat einen hohen Stellenwert. Die Versorgung aller chronisch Kranker beinhaltet die aktive Koordination zwischen Haus-, Fachärzten und anderen relevanten Gruppen. |
| --- | --- | --- | --- | --- |

**Teil 3d : Klinische Informationssysteme.** Die Verfügbarkeit aktualisierter Informationen über einzelne Patienten oder Patientengruppen ist ein entscheidendes Merkmal für die effektive Versorgung chronisch Kranker.

| **Spezifische Patientenregister (z.B. Liste von Patienten mit bestimmten Erkrankungen)** | …sind nicht vorhanden. | ..beinhalten Name, Diagnose, Kontaktdaten und das Datum des letzten Kontaktes entweder als klassische Karteikarte oder PC-Datenbank. | …ermöglichen die Abfrage und Gewichtung von Untergruppen (z.B. nach bestimmten klinischen Merkmalen). | …sind mit Leitlinien verknüpft und errinnern durch eine Reminderfunktion an notwendige Maßnahmen. |
| --- | --- | --- | --- | --- |

| **Reminder für Leistungserbringer** | …sind nicht vorhanden. | …beinhalten zum Zeitpunkt des Kontakts den Hinweis, dass bei diesem Patienten eine chronische Erkrankung vorliegt, beschreiben jedoch nicht ausstehende Behandlungsschritte/ Maßnahmen. | …beinhalten Informationen zu notwendigen Maßnahmen, die bei einer entsprechenden Patientengruppe in regelmäßigen Abständen durchgeführt werden sollten. | …beinhalten spezifische Informationen für das Team über die Einhaltung der Leitlinien zum Zeitpunkt des individuellen Patientenkontakts. |
| --- | --- | --- | --- | --- |

| **Feedback** | …ist nicht verfügbar oder nicht auf das Team zugeschnitten. | …ist unregelmäßig und unpersönlich verfügbar. | …erfolgt regelmäßig und in ausreichenden Abständen, um die Leistung einzuschätzen und ist auf das Team zugeschnitten. | …ist zeitnah und teamspezifisch. Es wird routinemäßig und persönlich von einem Mitarbeiter erstellt, um die Leistung des Teams zu optimieren. |
| --- | --- | --- | --- | --- |

| **Informationen über spezifische Patientengruppen** | …sind nicht verfügbar | …sind nur mit besonders hohen Aufwand verfügbar. | …sind auf Nachfrage, aber nicht routinemäßig verfügbar. | …werden für die Leistungserbringer routinemäßig angeboten, um bei der Erstellung eines Behandlungsplans berücksichtigt zu werden. |
| --- | --- | --- | --- | --- |

| **Behandlungspläne** | …werden nicht erstellt. | …werden durch ein standardisiertes Vorgehen erstellt. | …werden gemeinsam erarbeitet und beinhalten sowohl Aspekte des Selbst-Managements als auch Therapieziele. | ...werden gemeinsam erarbeitet und beinhalten sowohl Aspekte des Selbst-Managements, als auch ein klinisches Gesamtkonzept. Verlaufskontrollen finden statt und strukturieren die Versorgung. |
| --- | --- | --- | --- | --- |

| **Teil 4Vernetzung der Elemente des Chronic-Care-Modells.** Effektive Behandlungssysteme kombinieren alle Elemente des Chronic Care Modells z.B. die Verknüpfung der Ziele des Selbst-Managements mit Informationssystemen / Registern. |
| --- |

| **Informieren der Patienten über Leitlinien** | …findet nicht statt. | …findet auf Nachfrage oder durch allgemeine Veröffentlichungen statt. | …erfolgt durch spezifisches Informationsmaterial für jede Leitlinie. | …beinhaltet spezifische Materialien, die für Patienten entwickelt wurden, um ihre Rolle in der Erreichung der Leitlinien-Adherence zu beschreiben. |
| --- | --- | --- | --- | --- |

| **Informationssysteme/ Register** | …beinhalten keine Ziele des Patienten Selbst-Managements | …beinhalten Ergebnisse der ärztlichen Einschätzung des Patienten (z.B. Untersuchungsergebnisse, Bereitschaft sich im Selbst-Management zu engagieren), aber keine Zielvereinbarungen. | …beinhalten sowohl die Ergebnisse der ärztlichen Einschätzung des Patienten als auch die Ziele des Selbst-Managements, die gemeinsam mit dem Patienten entwickelt wurden | …beinhalten sowohl die Ergebnisse der ärztlichen Einschätzung des Patienten als auch die Ziele des Selbst-Managements, die gemeinsam mit dem Patienten entwickelt wurden. Zusätzlich beinhalten sie auch Reminder für Patienten und/ oder Leistungserbringer für die Verlaufskontrolle und für die regelmäßige Überprüfung der Zielvereinbarungen. |
| --- | --- | --- | --- | --- |

| **Kommunale Programme** | …gewährleisten kein Feedback an Akteure des Gesundheitssystem (z.B. Praxis, Krankenkasse) über Patientenfortschritte in ihren Programmen. | …gewährleisten ein unregelmäßiges Feedback (z.B. bei gemeinsamen Treffen) an die Akteure des Gesundheitssystems über Patientenfortschritte in ihrem Programm. | …gewährleisten ein regelmäßiges Feedback an die Akteure des Gesundheitssystems mittels standardisierter Prozesse (z.B. Internet basierte Verlaufsberichte). | …gewährleisten ein regelmäßiges, gemeinsam auf die Bedürfnisse des Patienten abgestimmtes Feedback an die Akteure des Gesundheitssystems, mit dem Ziel die Versorgungsangebote patientenorientiert weiterzuentwickeln. |
| --- | --- | --- | --- | --- |

| **Planung/ Organisation der Versorgung chronisch Kranker** | …beruht nicht auf einen bevölkerungsbezogenen Ansatz. | …nutzt allgemeine Daten (z.B. Routinedaten, DMP-Daten), um die Versorgung zu planen. | …nutzt allgemeine Daten, um die Versorgung proaktiv bevölkerungsbezogen zu planen. Dies beinhaltet die Entwicklung von Selbst-Management Programmen, sowie Zusammenarbeit mit kommunalen Anbietern. | …nutzt systematisch Daten und Eingaben des Praxisteams, um die Versorgung proaktiv bevölkerungsbezogen zu planen. Dies beinhaltet die Entwicklung von Selbst-Management Programmen, kommunale Zusammenarbeit einschließlich die Evaluation. |
| --- | --- | --- | --- | --- |

| **Terminvergabe für Verlaufskontrollen, Patienteinschätzung und Besprechung der Zielvereinbarungen** | …finden nicht statt. | …finden unregelmäßig statt. | …wird gewährleistet durch die Übertragung der Verantwortung an bestimmte Mitarbeiter (z.B. Medizinische Fachangestellte) | …wird gewährleistet durch die Übertragung der Verantwortung an bestimmte Mitarbeiter. Diese nutzen vorhandene Datenquellen, um mit Patienten und Praxisteam zu kooperieren (z.B. Register, Praxissoftware). |
| --- | --- | --- | --- | --- |

| **Leitlinien für die Versorgung chronisch Kranker** | …werden dem Patienten nicht zur Verfügung gestellt/ nicht gemeinsam genutzt. | …bekommen Patienten, die ein besonderes Interesse am Selbst-Management ihrer Erkrankung haben. | …werden für alle Patienten zur Verfügung gestellt, um Ihnen bei der Entwicklung von Programmen zum Selbst-Management oder zur Verhaltensänderung zu helfen und geben vor, wann der Patient einen Leistungserbringer aufsuchen soll. | …werden gemeinsam vom Patient und Praxisteam genutzt, um Ihnen bei der Entwicklung von Programmen zum Selbst-Management oder zur Verhaltensänderung zu helfen. Hierbei werden die individuelle Bereitschaft zur Verhaltensänderung und die Patientenziele berücksichtigt. |
| --- | --- | --- | --- | --- |
